# Network analysis of the relationship between negative life events and depressive symptoms in the left-behind children

**DOI:** 10.1186/s12888-021-03445-2

**Published:** 2021-09-01

**Authors:** Kuiliang Li, Yu Guang, Lei Ren, Xiaoqing Zhan, Xuejiao Tan, Xi Luo, Zhengzhi Feng

**Affiliations:** 1 School of Psychology, Army Medical University, Gao Tanyan, Sha Pingba, Chongqing, 400038 P.R. China; 2 Department of Gynecology, The First Affiliated Hospital of Shenzhen University, Shenzhen Second People’s Hospital, Shenzhen, 518028 P.R. China; 3 Shenzhen Dapeng New District Maternal and Child Health Hospital, Shenzhen, 518120 P.R. China; 4 Department of Military Medical Psychology, Fourth Military Medical University, No. 169 West Changle Road, Xi’an, 710032 P.R. China; 5 Department of Medical English, School of Basic Medical Sciences, Army Medical University, Gao Tanyan, Sha Pingba, Chongqing, 400038 P.R. China

**Keywords:** Left-behind children, Depression, Negative life events, Network analysis

## Abstract

**Background:**

There are 68.77 million left-behind children in China, who are at a great risk of depression associated with negative life events. Our study aims to investigate the most central symptoms of depression in left-behind children and the relationship between depressive symptoms and negative life events using network analysis.

**Method:**

A cross-sectional data set (*N* = 7255) was used, which included children and adolescents aged 7 to 17. Network analysis was used to evaluate: 1) the most central symptoms among the items included in Child Depression Inventory (CDI) of the left-behind children; 2) bridge symptoms between depressive disorder and Adolescent Self-Rating Life Events Check List (ASLEC) of the left-behind children; 3) differences in networks of depressive disorders between left-behind and non-left-behind children, and 4) differences in the network of depression and negative life events between left-behind and non-left-behind children. The stability and centrality indices of the network were also evaluated in the study.

**Results:**

The most central symptoms in the CDI among the left-behind children included self-hatred, crying, fatigue, and sadness. The items with the highest bridge strength centrality in the CDI-ASLEC network included academic stress, discrimination, and school performance decrement. Higher bridge strength values indicate a greater risk of contagion to other communities. The connections in the CDI-ASLEC network are denser in the left-behind children than in non-left-behind children.

**Limitations:**

The study which was conducted based on cross-sectional data shows that network analysis can only make undirected estimation, but not causal inferences.

**Conclusions:**

We identified the core symptoms of depression and the bridge symptoms between negative life events and depression in the left-behind children. These findings suggest that more attention should be paid to self-hatred, sadness, and fatigue in the treatment of depression in left-behind children. Intervention for academic stress and discrimination of the left-behind children may help to reduce the contagion of negative life events to depression symptoms.

## Introduction

China’s reform and opening-up and the subsequent social and economic development have led to the prosperity of many cities. A large amount of the population in the undeveloped areas in central and western China began to move to the developed area for employment opportunities. According to the Report on China’s Migrant Population Development, there were more than 200 million floating populations in China in 2020, and over 70% of them gathered in the developed region in eastern China. The data show that the average monthly income of the floating population is 4598 yuan. After paying for food, clothing, housing, and transportation, their disposable income is too low to afford their children’s education and housing in the developed region. Therefore, a large number of children are left behind by their parents in their hometown, who are known as left-behind children. The left-behind children are defined as children who have lived in undeveloped areas for more than 6 months and have one parent or both work in developed areas. In 2015, there were 68.77 million left-behind children in China, including 40.51 million in rural areas [1]. These children are usually taken care of by their relatives, especially grandparents, who pay more attention to their physiological needs rather than their psychological needs.

Although studies have shown that working in the developed area can increase family income and raise family status in the local area, it is rarely beneficial for left-behind children [2]. Researches show that left-behind children have more mental health problems than non-left-behind children [3–6], including a higher risk of depression [7], and anxiety [8], a stronger sense of loneliness [9], and higher suicide risk [10], highlighting severe psychological problems of these children and tough challenges posed by the problems.

Depression is the most common mental health problemin childhood [11], and depression in children and adolescents is usually the first episode of depression [12]. Early prevention and intervention can help reduce depression symptoms and the risk of recurrence [11, 13]. A previous systematic review and meta-analysis showed that left-behind children have higher depression risk and depression scores than non-left-behind children (RR 1.52 [95% CI 1.27–1.82]; SMD 0·16 [0·10–0·21]) [3]. However, at present, most studies only compare the differences in the score of depression scale between left-behind and non-left-behind children [14], while few studies have made a detailed comparison of depressive symptoms between left-behind and non-left-behind children. Therefore, it remains unknown whether the symptoms of depression differ between them.

Depression of children is associated with many factors, such as environmental stress [15], life trauma [16], bullying [17], etc. Studies show that children who are exposed to negative life events are at a higher risk of depression [18]. It is also reported that left-behind children are more likely to be exposed to negative life events. For example, in rural areas of Mexico, the children whose father works in other places have 39% higher incidence of disease than the non-left-behind children, and their incidence of diarrhea is increased by 51% [19]. In addition, the risk of physical and mental disorders in the left-behind children is higher than that in the non-left-behind children, such as car accidents, falls [3], sexual abuse, neglect [20] and bullying [21], and less communication with parents [6]. It is worth noting whether the negative life events experienced by left-behind children aggravate their depressive symptoms. Studies have shown that reducing these negative life events can alleviate depressive symptoms. For example, more frequent and longer parent-child communication can significantly reduce the incidence of depression in left-behind children [18]. Therefore, it is significant to investigate the effect of negative life events on the depression of the left-behind children.

We found in our previous studies that the overall prevalence of depression in the left-behind children was 24.8%. Meanwhile, we also found that high income, frequent parent-child communication, telephone communication or talking about learning experiences, school life and emotional experiences are protective factors for depression [22]. In addition, prevalence of depressive symptoms is higher in the left-behind children than in non-left-behind children, and negative life events are risk factors for depression in left-behind children [18]. Despite the work we have done above, it is still unclear which negative life event plays a major role in certain type of depressive symptom.

Few studies have investigated the relationship between negative life events and depression symptoms of left-behind children, and to our knowledge, no studies have been conducted to explore their relationship using network analysis. Therefore, the understanding remains limited regarding their relationship, as well as the major types of the negative life events experienced by the left-behind children and their core depression symptoms. This study aims to explore the relationship between negative life events and characteristics of depression symptoms of left-behind children using network analysis. The network usually consists of symptoms (nodes) of mental disorders and connections (edges) between them [23]. According to the network theory, diseases are usually caused by the interaction between symptoms, and the symptoms themselves are not mental diseases, but components of mental diseases [24]. Alleviating the symptoms of disease plays an important role in disease treatment. Targeting the core symptom derived from network analysis helps us treat the underlying disease [25].

We investigated the network structure of depressive symptoms of a large sample (*N* = 2517) of left-behind children and explored the relationship between depression and negative life events using network analysis. We aimed to: a) construct depression networks to identify the main symptoms of depressive disorders of left-behind children; b) compare the differences in depression networks between left-behind children and non-left-behind children; and c) use bridge centrality to identify the disease pathway linking negative life events and depressive symptoms of left-behind children.

In this study, two networks were constructed: the network of depressive symptoms and the network of both depressive symptoms and negative life events. These networks were used to identify the most central symptoms of depression disorders of left-behind children, to identify the negative life events with a stronger correlation with depressive symptoms of left-behind children using bridge symptoms, and to compare the differences in the two networks between left-behind children and non-left-behind children.

## Methods

### Ethics statement

The current study was reviewed and approved by the Medical Ethics Committee of the Department of Medical Psychology, Army Medical University (No. CWS20J007). The study was conducted in accordance with the Declaration of Helsinki guidelines. The approval from officials of sampled schools was obtained in a written form. Parents or legal guardians were contacted to provide consent on behalf of the children to participate in the study. After reading the informed consent, participants can complete the following survey if they want to further participate in this study. The participants were also assured that the survey was anonymous and personal information would not be disclosed.

### Participants

**Fig. 1:**
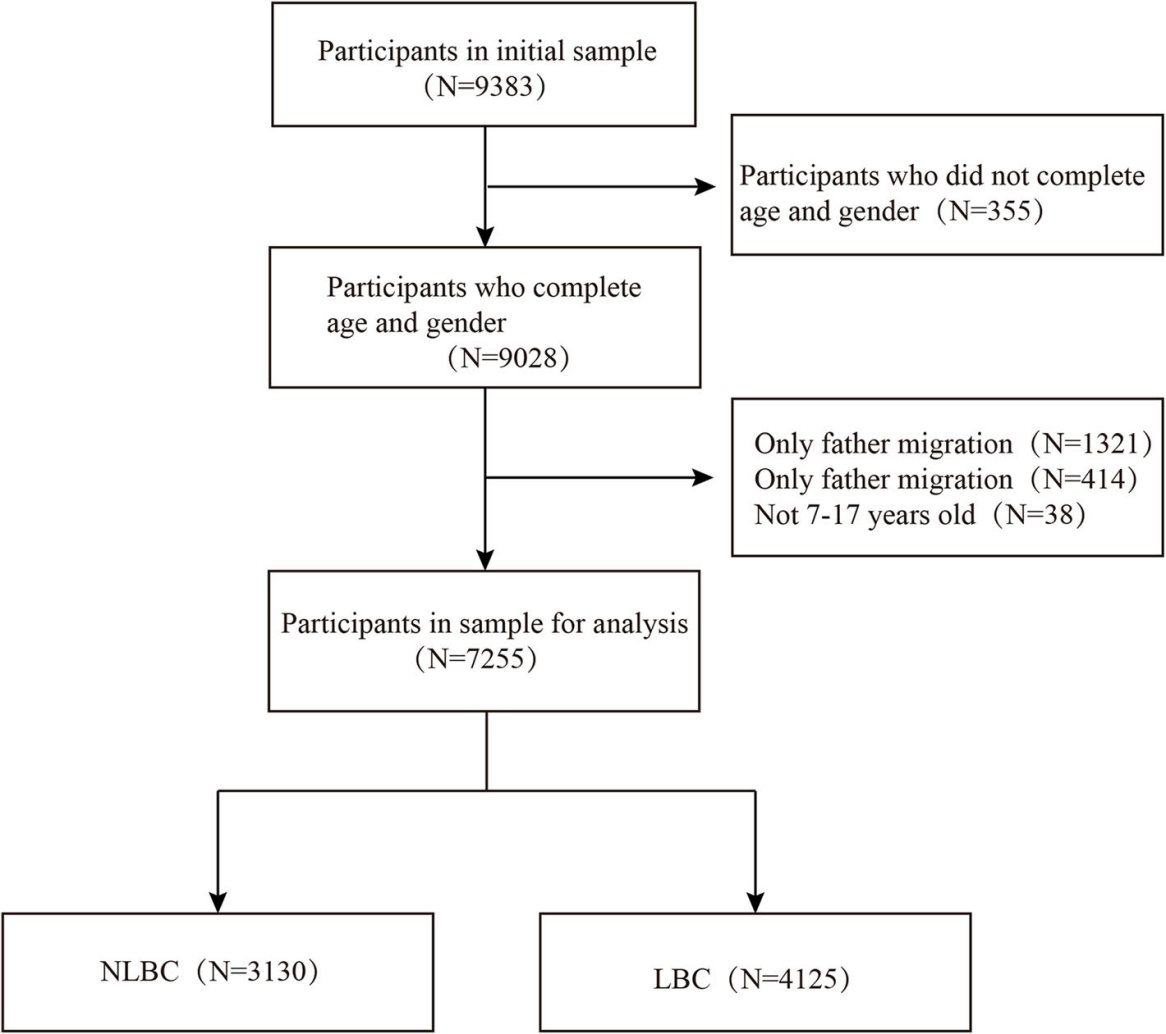
Flow of participants in the study]

This cross-sectional study using a dataset from a previous study [18] conducted a three-phase survey in districts and counties of Chongqing. In this study, we selected samples from 19 districts and counties: Chengkou, Wuxi, Wushan, Fengjie, Yunyang, Liangping, Fengdu, Dianjiang, Changshou, Shizhu, Pengshui, Qianjiang, Wulong, Youyang, Xiushan, Tongnan, Dazu, Hechuan and Jiangjin, with a total of 9383 participants. All participants completed the Child Depression Inventory (CDI) and the Adolescent Self-Rating Life Events Checklist (ASLEC), as well as the demographic information questionnaire. Left-behind children were selected according to three criteria: 1) Both parents have been working outside the home for more than 6 months; 2) They live with family members other than their parents; and 3) They are aged between 7 and 17 years old. Participants were excluded if they missed responses to more than 50% items or had a single parent going out. Only 7255 children were included at last. Figure 1 shows the flow of participants for this study.

### Assessments

#### Child depression inventory (CDI)

The depressive symptoms of left-behind children were determined based on the Chinese version of CDI [26], which is the most commonly used questionnaire for assessing depression among children and adolescents aged 7–17 [27]. CDI is a self-report questionnaire of 27 items on five dimensions, which is designed to assess the loss of pleasant sensation (e.g. symptoms like unhappiness), inefficiency (e.g. less motivation), interpersonal problems (e.g. unable to maintain relationships), negative emotions (e.g. sadness), and negative self-assessment (e.g. feeling of being unable to do anything) [28]. Each item has three statements of different degree and requires the child to choose the one that best describes himself or herself in the past 2 weeks. Each item is scored on a scale of 0 (low), 1 (medium) and 2 (high), with a total score ranging from 0 to 54. Half of the items are scored in reverse order, with higher scores indicating more severe depressive symptoms. The generally recommended cut-off point for depression is 19 or 20 points [28]. However, the cut-off point in this study was 12 points as this study was not aimed to diagnose depression, and a lower cut-off point could include more likely depressed individuals [29]. In the current study, the α coefficient of the questionnaire was 0.83, showing good consistency.

#### Adolescent self-rating life events check list (ASLEC)

ASLEC was developed by Liu Xianchen et al. [30] to assess the frequency and stress intensity of stressful life events among adolescents, especially middle school students. The scale consists of 27 items on six dimensions, i.e. interpersonal relationship, academic stress, punishment, loss, health adjustment and others. ASLEC is a self-rating scale, requiring participants to determine whether the event described in each item has occurred within the past 3 months; if so, they need to tick on a scale of 1 (no influence) to 5 (significant influence), and if not, to tick the box of “not happening” scored as “no influence”. The total score of the scale ranges from 27 to 135, with higher scores indicating greater total stress. The scale shows good reliability and validity. In the current study, the α coefficient of the scale was 0.927.

#### Data analysis

##### Missing data

All data were analyzed in R 4.0.0. Using the mice software package in R language, we adopted the insertion method for the data with less than 50% missing individual-level data [31]. In this study, there were missing data for 3754 options, accounting for 0.95% of the total options (391,770).

##### Glasso network

The EBIC glasso function of the qgraph software package in the R language was used to estimate the two networks [32], both of which used datasets (*N* = 7255). GGM was regularized by graphical LASSO (Least Absolute Shrinkage and Selection Operator) algorithm. This can shrink all edges and make small edges become zero-weight edges to obtain a more stable and interpretable network. The GGM adjustment parameter was set to the recommended value of 0.5 to well judge and measure the sensitivity and specificity of discovering true edges [33]. In the visual network, the red edge represented the negative partial correlation between nodes, and the blue edge represented the positive partial correlation between nodes, with thicker edges indicating the stronger correlation between nodes. We estimated the network containing depressive symptoms (i.e. CDI network), and the depression network containing negative life events (i.e. CDI-ASLEC network).

##### Stability and accuracy analysis

The stability and accuracy of the network were calculated with the bootnet function of R software [34]. First, the accuracy of the edge weights was evaluated by calculating 95% confidence intervals (CI) using bootstrapping. The narrower the CI was, the more accurate the estimation of the edge weights and the centrality index would be. The stability of the centrality index was then estimated by calculating the correlation stability coefficient using case-dropping bootstrapping. The correlation stability coefficient (CS-coefficient) is the maximum percent of cases that can be excluded if the correlation between the centrality indices of the original sample and the subset of the sample is 0.70 or higher (95% probability). The CS-coefficient (how much data can be discarded) should not be less than 0.25, preferably higher than 0.50 [32].

##### Centrality and difference analysis

The qgrath package of R software was used to calculate the centrality index [35], and the bootnet package was used to test the node centrality difference [34]. Studies have shown that strength centrality is more stable than compactness and intermediation [36, 37]. Therefore, strength centrality was used as an index in this study. Strength centrality represents the sum of edge weights of each node (e.g. correlation coefficients), reflecting the possibility that the activation of a certain symptom may lead to the activation of others [24].

##### Bridge symptoms

Bridge symptoms are thought to be the overlapping symptoms of two psychiatric disorders [38]. In this study, we used bridge centrality statistics to determine the overlapping symptoms of negative life events with depressive symptoms [39]. Bridge strength centrality is the best index to identify bridge nodes, so we calculated bridge strength centrality and bridge expected influence centrality. According to the report by Jones et al., eliminating bridge symptoms can prevent the spread of one disease to another.

##### Comparison between left-behind children and non-left-behind children

In order to compare the differences in depression network between left-behind children and non-left-behind children, we used the R “network contrast test (NCT)” package. NCT uses permutation test to compare the invariance in global strength (i.e. the sum of all edge weights) and structure between two networks [40]. In order to compare the differences between the two samples, we generated a depression network and a depression-negative life event network to explore whether their depressive symptoms share the same correlation, whether their strongest symptoms are consistent, and whether they experience the same negative life events.

## Results

The left-behind children accounted for 56.86% (*N* = 4125) and non-left-behind children accounted for 43.14% (*N* = 3130) of the total sample (*N* = 7255). The cut-off value for depressed individuals was 12 points in the depression scale. The final analysis included 2517depressed left-behind children and1716 depressed non-left-behind children. More information on age, gender, and depression level can of the sample can be found in Table 1**.**

**Table 1:** Demographics and depression scores of the sample (*N* = 7255)

Variables	LBC	NLBC
Total	4125	3130
Age (year)		
7–9	851	667
10–12	1167	1010
13–15	1372	869
16–17	735	584
Gender		
Female	1992 (48.3%)	1664 (53.2%)
Male	2133 (51.7%)	1466 (46.8%)
Ethnicity		
Han	3462	2516
Minority	600	585
Not Reported	56	25
Depression Scores> 12	2517	1716

### Network stability

The stability of edge weights of the two networks met the requirements, greater than the recommended 0.5 [32]. A higher CI of edge weights indicated a higher accuracy of network estimation. The edge weight of network 1 and network 2 was 0.59 and 0.75, respectively. In addition, since the strength centrality index was previously reported to have higher repeatability and stability [32], we focused more on the strength centrality index. The CS-coefficient of strength centrality index in network 1 and network 2 was 0.594 and 0.75, respectively, both higher than the recommended critical value of 0.25 [41].

### Centrality

#### Network 1 (CDI network)

**Fig. 2:**
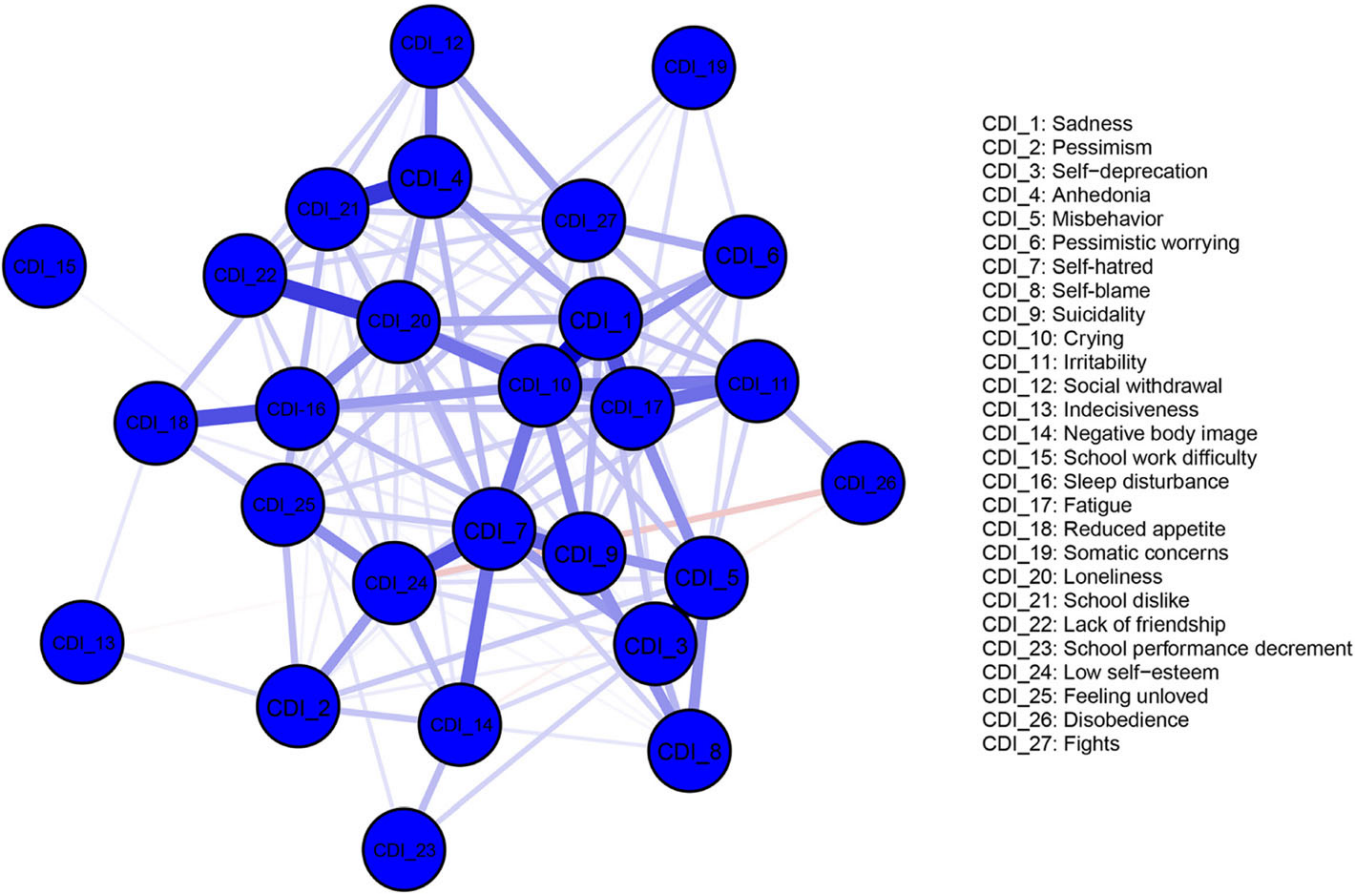
An estimated network model for depressive symptoms in the total sample (*N* = 2517)]

**Fig. 3:**
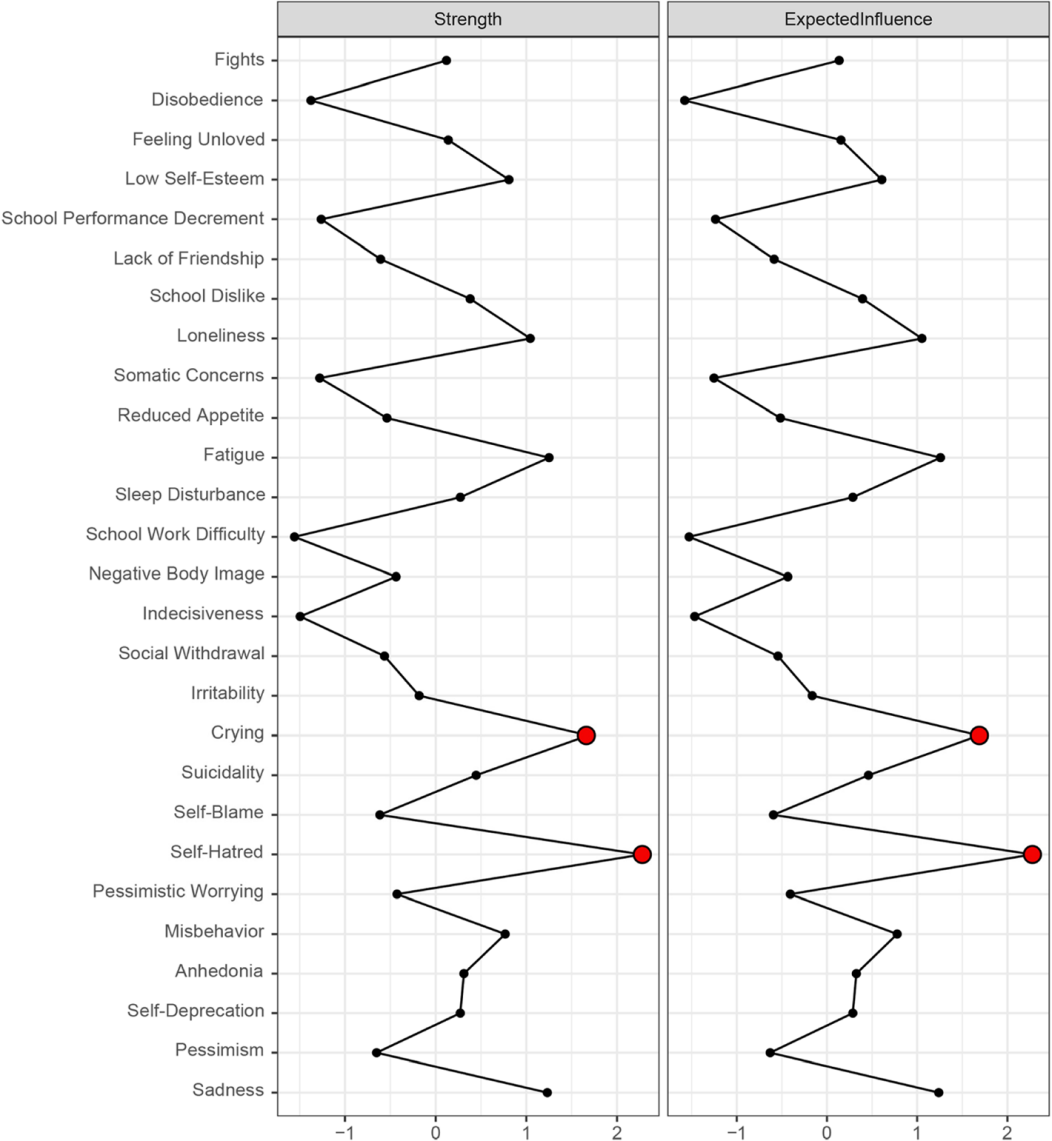
Centrality plot of the depressive symptoms, shown as standardized values z scores]

The structure and centrality of depression network was shown in Fig. 2 and Fig. 3, respectively. Network 1 only contained 27 central depressive symptoms, and the symptoms with the highest strength centrality included self-hatred (S = 2.28), crying (S = 1.68), fatigue (S = 1.25), and sadness (S = 1.23). The results of the strength centrality difference test showed that the estimated strength centrality of self-hatred was significantly higher than that of other symptoms (*P* < 0.05).

#### Network 2 (CDI-ASLEC network)

**Fig. 4:**
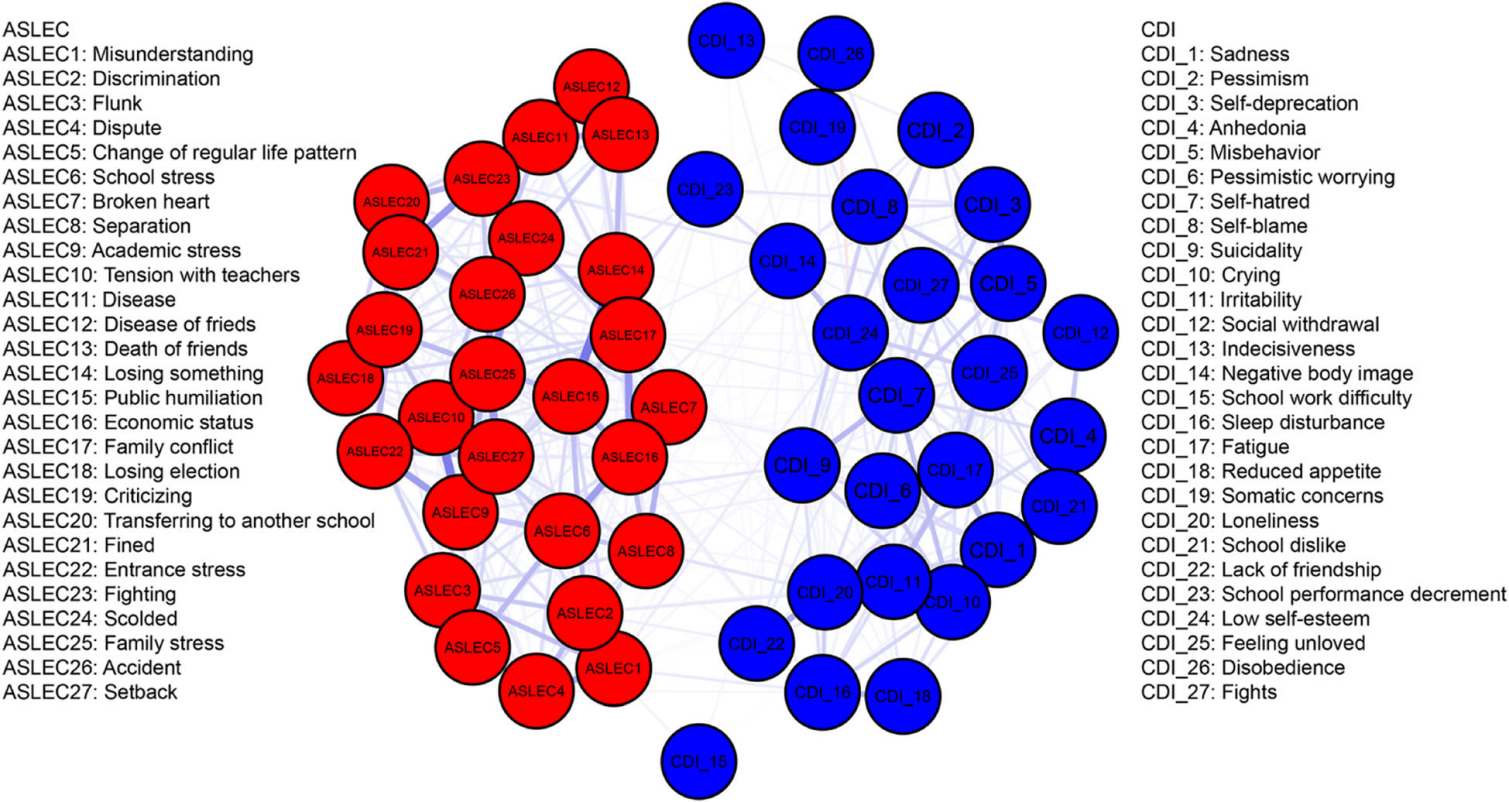
An estimated network model for negative life events and depressive symptoms in the total sample (*N* = 2517)

**Fig. 5:**
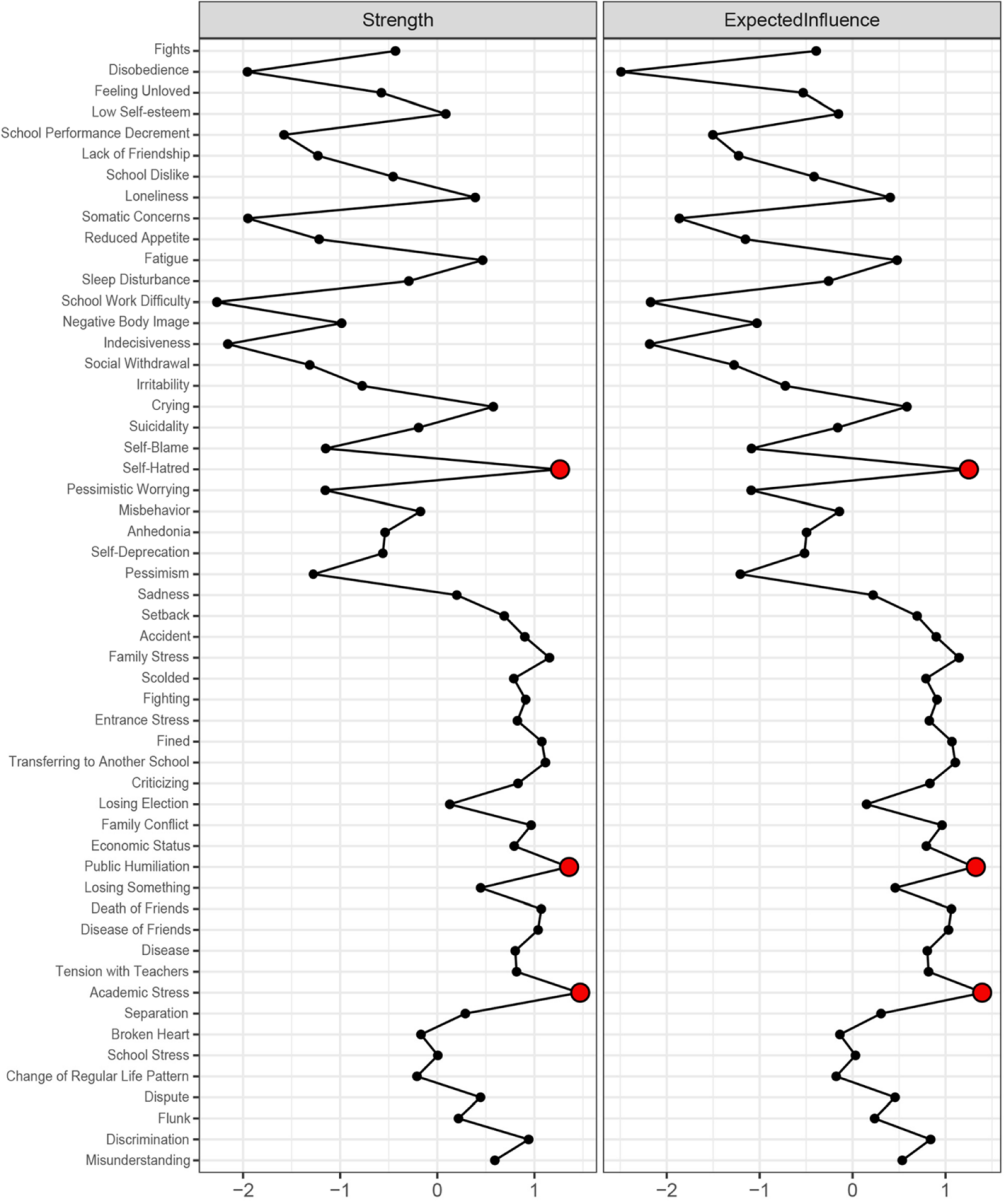
Centrality plot of the negative life events and depressive symptoms, shown as standardized values z scores

The structure and centrality of the depression-adolescent life event network was shown in Fig. 4 and Fig. 5, respectively. The network included depressive symptoms and negative life events, and the symptoms with the highest strength centrality were academic stress (S = 1.46), public humiliation (S = 1.35), and self-hatred (S = 1.24). The results of the strength centrality difference test showed that the estimated strength centrality of self-hatred was significantly higher than that of other symptoms (*P* < 0.05).

#### Bridge symptoms

##### Network 2

There were some bridge symptoms between depression and negative life events. The symptoms with the highest bridge strength centrality were: academic stress (BS = 0.15), discrimination from others (BS = 0.14) and school performance decrement (BS = 0.12) due to depression. The symptoms with the highest bridge expected influence centrality included academic stress (EI = 0.14) caused by negative life events and school performance decrement (EI = 0.12) due to depression.

##### Comparative analysis of networks

NCT results showed that there was no significant difference in network invariance (M = 0.08, *p* > 0.05) and global strength invariance (S = 2.51, LBC = 5.34, NLBC = 2.83, *p* > 0.05) in the CDI network between depressed left-behind children and non-left-behind children. The strength centrality index of the two networks was highly correlated (*r* = 0.85). In the CDI-ASLEC network, there was no significant difference in the network invariance between left-behind children and non-left-behind children (M = 0.09, *p* > 0.05), but there was a significant difference in the global strength invariance (S = 4.61, LBC = 18.99, NLBC = 14.38, *p* < 0.01), indicating that differences exist in the global connection of symptoms, but not in the interaction between symptoms.

## Discussion

We used network analysis to study the characteristics of depression network and depression-negative life events network with a large sample (*N* = 2517) of left-behind children. We found that differences existed in the strength of symptoms in the CDI network of left-behind children, and self-hatred has the highest centrality in the network, consistent with the previous research which shows that self-hatred also has the highest centrality in the CDI of the non-left-behind children [42]. This means that left-behind and non-left-behind children have similar central symptoms in the CDI depression network. NCT analysis in our study also shows that there is no significant difference in the CDI depression network between left-behind and non-left-behind children. These findings may be explained by the fact that left-behind and non-left-behind children share the same developmental stage, such as puberty. On the one hand, the left-behind children begin to have self-awareness [43], and the derived self-related internal information [44] plays an important role in depression [45]. On the other hand, with the development of self-identity in adolescence, the children tend to pay more attention to self-achievements, family atmosphere, etc. [46]. Indeed, previous studies have shown that lower self-worth is associated with depression [47]. The negative self-information has become a potential risk factor of adolescent depression [48], while positive self-identity information can prevent negative effects [49]. Last but not least, depression may also be associated with physical development during puberty, such as the development of secondary sexual characteristics and height [50], and with excessive attention paid to appearance [51].

In addition, we also found some other high-intensity symptoms, such as crying, fatigue and sadness. Among them, crying and fatigue are not the most central symptoms according to the previous report [42]. Our finding about fatigue and sadness can be explained by the fact that left-behind children experience more bullying or abuse [52], and they can only reduce the risk of depression through self-sympathy [21]. Fatigue symptoms stem from more manual labor they need to shoulder when living with grandparents. Compared with non-left-behind children, left-behind ones reported increased working hours [53], and the fatigue increased the risk of depression [54, 55]. Therefore, we should improve the social support for left-behind children, including protecting them against abuse and bullying and reducing their extra working hours, to reduce their risk of depression.

It is interesting to find that these central symptoms in the depression network of the left-behind children differed from those in CDI-ASLEC network which included the negative life events. In CDI-ASLEC network, academic stress and public humiliation in ASLEC and school performance decrement in CDI have the highest centrality reflecting great academic stress and impaired self-esteem of the left-behind children. It should be noted that these networks are undirected rather than directed (causal networks). The most central symptoms were not obtained based on all symptoms, but on the symptoms we input.

We also identified bridge symptoms in CDI-ASLEC network. Bridge symptoms are considered to be an illness pathway for one disorder to spread to another. Therefore, when one disorder appears, intervention of potential bridge symptoms can effectively prevent the spread of disorders and the development of complications [39]. In this network, academic stress, discrimination and school performance decrement have the strongest bridge strength centrality in the left-behind children, consistent with the previous studies. Parents’ inquiry about study accounts for a high proportion of their communication with their children [18], which increases the children’s academic stress, and in turn increases the risk of depression [56]. In addition, the self-esteem of left-behind children will be impaired by discrimination by others [57], and low self-esteem is more likely to lead to depression [58]. Taking corresponding measures to intervene bridge symptoms, especially those with high intensity centrality, can effectively reduce the risk of depression. For example, some studies have reported that regulating self-esteem bridge symptoms can reduce the impact of negative life stress on depression [57]. However, future studies are warranted to confirm if academic pressure and difficulties can serve as targets for intervention.

Finally, we used the network comparison test to compare the differences between left-behind children and non-left-behind children in CDI network and CDI-ASLEC network. It is found that, firstly, in CDI network, there is no difference in network structure and global strength between left behind and non-left behind children; secondly, in CDI-ASLEC network, there is no difference in network structure between the left-behind and non-left-behind children. However, there is difference in global strength, indicating that the characteristics of left-behind children network have stronger connection than those of the non-left-behind children. These results show that compared with non-left-behind children, negative life events have a greater impact on left-behind children. One reason is that left-behind children are exposed to more negative life events; another reason is that left-behind children show more depressive symptoms when they encounter negative life events than non-left-behind children [18]. Therefore, stress caused by the negative life events is a common risk factor for depression in the left-behind children.

In short, this study uses network analysis to obtain more subtle results. Our previous research found that negative life events are a risk factor for depression, and proper and adequate communication between the left-behind children and their parents can effectively reduce the impact of negative life events on depression [18]. This study further explored the relationship between negative life events and depression from the perspective of symptoms. The results regarding bridge symptoms indicate that academic stress and school performance decrement are the main pathways linking negative life events and depression. This result further supports our previous findings that the parent-child communications about learning experiences and school life are protective factors for depression [22]. When communicating with the left-behind children, the parents should avoid increasing their academic stress, and meanwhile, they should show more care about their study and life. By doing so, they can reduce the depression of left-behind children caused by negative life events more effectively.

### Limitations

There are some limitations in this study. First, we used sample data from the results of a cross-sectional survey, thus we cannot infer the causality dynamically. Second, the data is from previous research, with a poor timeliness. Therefore, it is necessary to repeat the investigation within a certain period of time to explore the evolvement of depressive symptoms of the left-behind children. In addition, due to the limitations of the study method itself, we did not input all the symptoms into the network model while other surveys, using different survey tools, found network structures differ from those of CDI [59, 60].

## Conclusions

This study explores the characteristics of CDI network and CDI-ASLEC network of the left-behind children aged 7–17 using network analysis. The results show that negative life events of left-behind children are closely associated with depression. Besides self-hatred, attention should also be paid to sadness and fatigue in the treatment of depression of the left-behind children. The intervention of academic stress and discrimination by others should also be considered in the treatment of depression, which may help alleviate the effect of negative life events on depression. The identification of core symptoms of depression facilitates the diagnosis and treatment of depression, offers suggestions for the reform of public policies for the left-behind children, and provides guidance on the education of their children for parents and guardians.

## Data Availability

The datasets generated and/or analyzed during the current study are not publicly available due to the privacy of participants but are available from the corresponding author on reasonable request.

## Abbreviations

LBC: Left behind children
NLBC: Non-left behind children
CDI: Child depression inventory
ASLEC: Adolescent self-rating life events checklist
CI: Confidence interval
CS: Correlation stability
GGM: Gaussian graphical model
LASSO: Least Absolute Shrinkage and Selection Operator
NCT: Network contrast test
